# Genetic Variation among *Heterodera schachtii* Populations Coincided with Differences in Invasion and Propagation in Roots of a Set of Cruciferous Plants

**DOI:** 10.3390/ijms24076848

**Published:** 2023-04-06

**Authors:** Rasha Haj Nuaima, Holger Heuer

**Affiliations:** Institute for Epidemiology and Pathogen Diagnostics, Julius Kühn Institute (JKI)-Federal Research Centre for Cultivated Plants, Messeweg 11-12, 38104 Braunschweig, Germany

**Keywords:** cruciferous hosts, *Heterodera schachtii*, populations, *vap1* (venom allergen-like protein gene)

## Abstract

Genes of host plants and parasitic nematodes govern the plant–nematode interaction. The biological receptors and parasitism effectors are variable among plant species and nematode populations, respectively. In the present study, hatch testing and bioassays on cabbage, oilseed radish, and mustard were conducted to compare the biological characteristics among six populations of the beet cyst nematode *Heterodera schachtii*. Genetic patterns of the *vap1* gene for the studied populations were distinct as shown by denaturing the gradient gel electrophoresis of PCR-amplified gene fragments. Concurrently, significant differences in the hatching rates, number of penetrated J2 in roots, and eggs/cyst ratios among the six nematode populations for the three cruciferous species were observed. In conclusion, analyzing the population genetic structure of *H. schachtii* plays a pivotal role in illustrating the variability in the plant–nematode interaction among its populations and plant species, which in its role leads to developing nematode management depending on plant resistance.

## 1. Introduction

Populations of plant-parasitic nematodes (PPNs) differ in their intraspecific genotypes and pathotypes [1,2,3,4,5,6,7,8,9], complicating nematode management since each population may require appropriate measures adjusted to its specific virulence.

Among the PPNs that showed high genetic variability within and among its geographical populations is the beet cyst nematode *Heterodera schachtii* [5,7]. This nematode causes significant losses in the production of sugar beet and other crops from 23 plant families [10,11]. Rotation with non-host crops, the cultivation of nematode-resistant or tolerant sugar beet cultivars, and cover cropping with resistant varieties are essential measures to control this nematode [10]. Reducing the population density below the economic threshold determines the effectiveness of these plant species and cultivars. Hence, the management of *H. schachtii* depends on plant genes that limit nematode propagation, either by providing an unsuitable environment in the case of non-host plants or through resistance genes. However, because of the genetic variability among *H. schachtii* populations [5,7], the plant–nematode interaction will occur in a population-specific manner. A resistant variety may reduce the nematode density of some populations without affecting others [4]. An in-depth study of population characteristics will therefore provide suggestions for plant breeders to produce resistant varieties that are specialized, effective, and more sustainable toward PPNs. In addition, some agricultural practices such as the passive transfer of PPNs, plowing, and the continuous cultivation of the resistant cultivar affect the genetic structure of PPNs [7]. Monitoring the characteristics of nematode populations will help to limit those measures that lead to the spread of virulent genes and that increase the adaptability of nematode populations [7].

Results of the study published by Nuaima et al. (2019) illustrated the difference in the profiles of the parasitism gene *vap1* among *H. schachtii* populations originating from different regions in Germany [7].

The venom allergen-like proteins (VAP) are members of the SCP/TAPS group, which includes a wide range of proteins found in plants and animals such as nematodes [12].

*vap1* was expressed in the J2 of cyst nematodes during root penetration and migration [13,14]. The secreted effector protein VAP1 interacts with the immunity system of the host plant and exhibits high genetic variation within and among populations of cyst nematodes [5,14,15].

The DGGE profiles of *vap1* were distinct between populations of *H. schachtii* that differed in the penetration ratio of their second-stage juveniles (J2) [16]. Moreover, the pathotypes of the potato cyst nematodes (PCN) have variable genetic patterns of *vap1* [6].

To this point, investigating the plant–nematode interaction of the genetically variable populations of *H. schachtii* will provide insights into the relatedness between their biological and genetic characteristics

In this study, we aimed to characterize the biological traits of six genetically different field populations of *H. schachtii* originating from three federal states in Germany. Hatching percentages, penetration rates of second-stage juveniles (J2), and ratios of eggs per cyst propagated on three cruciferous species were evaluated. To confirm the genetic difference among the six nematode populations, *vap1* gene profiles were analyzed using denaturing gradient gel electrophoresis (DGGE) of PCR-amplified gene fragments.

## 2. Results

### 2.1. Egg Hatch of H. schachtii Populations

**Figure 1:**
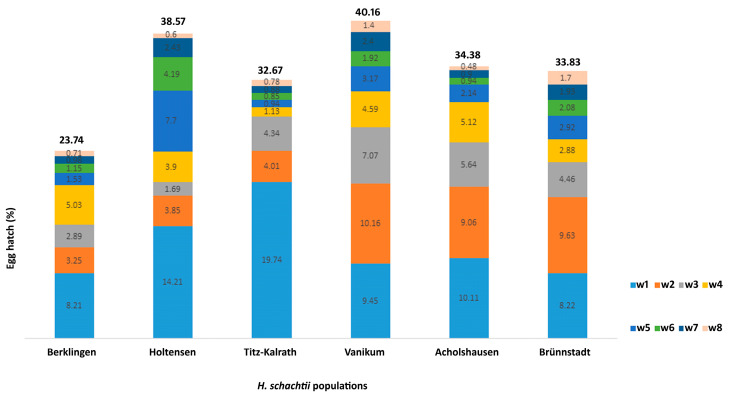
Egg hatching percentages of *H. schachtii* populations in zinc chloride solution over eight weeks. Each color represents the percentage ratio of J2 collected in the corresponding week to the egg content of 500 cysts. The percentage above each column refers to the total hatch percentage for each population during the eight weeks.

Within the population, the percentage of hatched eggs was the highest after seven days of placing the cysts in Zncl2 for Berklingen, Holtensen, Titz-Kalrath, and Acholshausen, and after 14 days for Vanikum and Brünnstadt (Figure 1). For each population, the percentage of hatched eggs after the fifth, sixth, and seventh weeks was lower than after the third and fourth weeks, except for the population of Holtensen, whose hatching percentage was 7.7%, 4.2%, and 2.4% after the fifth, sixth, and seventh week, respectively, and 1.7% and 3.9% after the third and fourth week, respectively. The percentage of hatched eggs was at its lowest after the eighth week for all populations.

Among the populations, the Titz-Kalrath and Berklingen had the highest and lowest number of hatched eggs after the first week, respectively. After the second and third weeks, the number of eggs hatched in the populations of Vannikum, Acholshausen, and Brünnstadt was higher than in the others. After the fourth week, the hatching rate was the highest for the Berklingen and Acholshausen populations (Figure 1).

The total number of hatched eggs was the highest in the Vanikum and the lowest in the Berkling population. Since the eggs of each population had their timeline for hatching, there was no specific paradigm for the relationship between the time and the hatching rate that might be predicted (Figure 1).

### 2.2. Penetration of H. schachtii Populations into the Roots of Three Cruciferous Species

The six populations of *H. schachtii* differed in their penetration rates into the roots of three host plants after seven days of inoculation.

**Figure 2:**
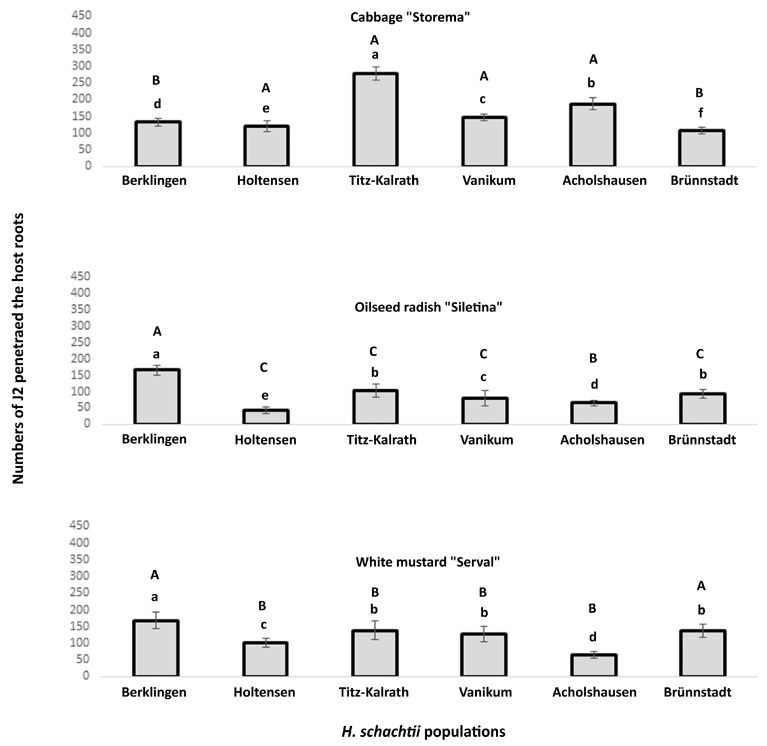
Numbers of second-stage juveniles (J2) from six populations of *H. schachtii* that penetrated roots of the susceptible cabbage (*Brassica oleracea* L., cv. Storema) or susceptible oilseed radish (*Raphanus sativus* L., cv. Siletina) or resistant white mustard (*Sinapis alba* L., cv. Serval). In total, 450 J2 were added to a seven-day seedling grown in the loess substrate and incubated under greenhouse conditions (20/16 °C for a 16/8 h day/night cycle) for seven days. The counting was for J2 in the roots, stained by acid fuchsin. (a,b,c,d,e,f) compare the mean numbers of J2 among populations that penetrated the same plant species. (A,B,C) compare the mean numbers of J2 of the same population that penetrated the three plant species. Different letters indicate significant differences (Wilcox’s test, *p* < 0.05, *n* = 8).

Significant differences in the number of J2 penetrating the susceptible cultivar of cabbage (*Brassica oleracea* L., cv. Storema) appeared among the six populations. The highest penetration rate was observed for Titz-Kalrath and the lowest for the population of Brünnstadt (Figure 2).

The difference between the populations in the numbers of penetrated J2 was less for roots of oilseed radish (*Raphanus sativus* L. var. Siletina) than for cabbage. Significant differences appeared between 14 pairs of population comparisons, while no significant difference was determined between the Titz-Kalrath and Brünnstadt populations (Figure 2). The Berklingen and Holtensen populations showed the highest and the lowest numbers of J2 penetrating the roots of Siletina, respectively (Figure 2).

The penetration rate into the roots of the resistant cultivar of white mustard (*Sinapis alba* L. var. Serval) varied among the 12 pairs of population comparisons. The Titz-Kalrath, Vanikum, and Brünnstadt populations were similar in the penetration rate of their J2. The Berklingen and Acholshausen populations correspondingly showed the highest and lowest numbers of J2 penetrating the roots of the Serval (Figure 2).

Differences in the counts of penetrated J2 appeared also within the same population among the three plant species. Each population of Holtensen, Titz-Kalrath, Vanikum, and Brünnstadt varied in their penetration among the three plant species. For the Berklingen and Acholshausen, the numbers of J2 penetrating roots of Siletina and Serval were similar (Figure 2). This result indicated that each *H. schachtii* population has a specialized interaction with each plant variety.

### 2.3. Propagation of H. schachtii Populations on Three Cruciferous Species

**Figure 3:**
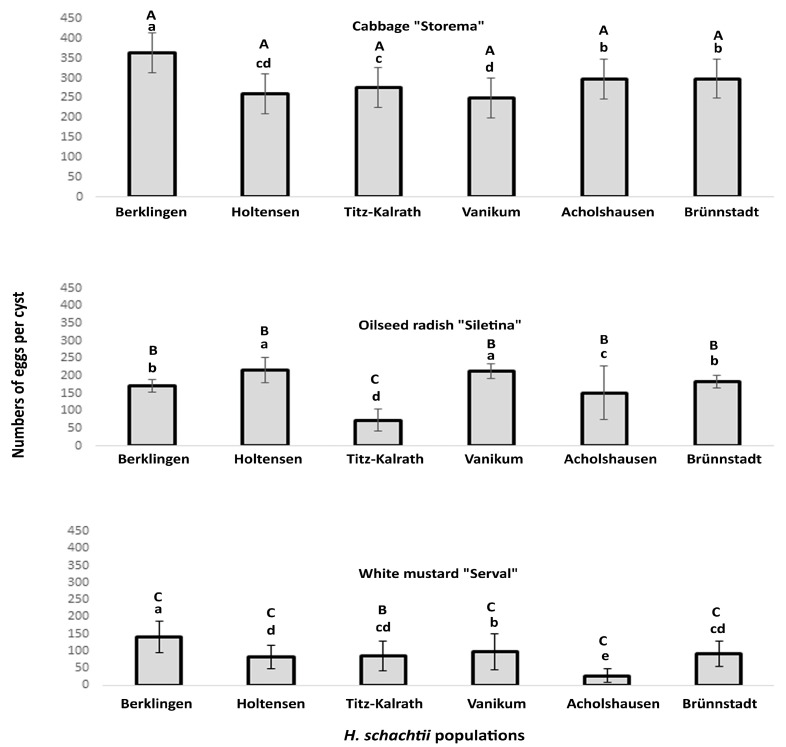
Numbers of eggs per propagated cyst of six populations of *H. schachtii* reared for two nematode generations on the susceptible cabbage (*Brassica oleracea* L., cv. Storema) or susceptible oilseed radish (*Raphanus sativus* L., cv. Siletina) or resistant white mustard (*Sinapis alba* L., cv. Serval). For each two-week seedling, 450 J2 were added and incubated under greenhouse conditions (20/16 °C for a 16/8 h day/night cycle) for three months. The counting was for eggs contained in the cysts extracted from the loess substrate by floatation on MgSO_4_ solution (1.28 specific density). (a,b,c,d,e) compare the mean numbers of egg/cyst ratio among populations reared on the same plant species. (A,B,C) compare the mean numbers of egg/cyst ratio of the same population reared on the three plant species. Different letters indicate significant differences (Wilcox’s test, *p* < 0.05, *n* = 8).

The egg content per cyst resulting from the propagation of *H. schachtii* on cabbage/Storema, oilseed radish/Siletina, and mustard/Serval for two generations was significantly different among geographically different nematode populations (Figure 3).

On cabbage, the egg count was highest in the Berklingen population and lowest in Vanikum.

Populations from the same region showed a variable ratio of eggs per cyst. The Lower Saxony or North Rhine Westphalia populations differed in their egg counts; however, the Bavarian ones were similar in this parameter (Figure 3). Despite belonging to geographically different regions, individual populations were similar in their pathogenicity, as in the Holtensen/Lower Saxony and North Rhine Westphalia populations (Figure 3).

On oilseed radish, the egg count was highest in the Holtensen and Vanikum populations and lowest in the Titz-Kalrath one (Figure 3). This measure was different between populations of the same region but similar for different ones, as the populations of Holtensen and Vanikum from Lower Saxony and North Rhine Westphalia, correspondingly.

On mustard, the egg count was highest in the Berklingen population and lowest in the Acholshausen one. The difference in the ratio of eggs per cyst did not correlate to the origin region. For example, the Lower Saxony populations differed in the number of produced eggs, however, the Titz-Kalrath/North Rhine Westphalia and Holtensen/Lower Saxony populations were similar in their egg content.

Overall, for all populations, the lowest eggs/cyst ratio was on the resistant mustard and the greatest on the susceptible cabbage, except the Titz-Kalrath population, whose propagated eggs were higher on mustard than on oilseed radish.

### 2.4. Variability of the Parasitism Gene vap1 among H. schachtii Populations

**Figure 4:**
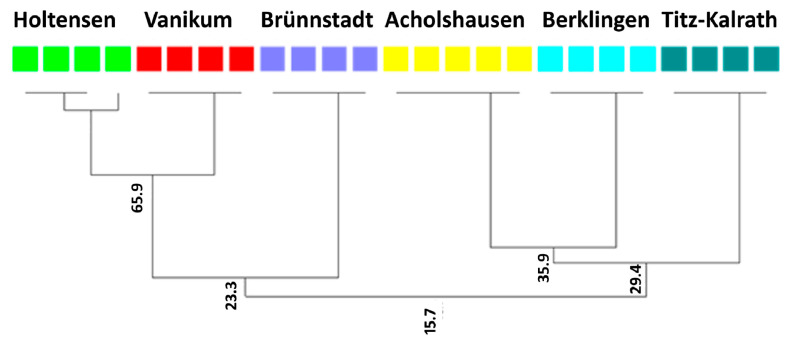
UPGMA clustering based on the Jaccard similarity coefficient, showing the genetic differences in *vap1* patterns among six populations of *H. schachtii* based on DGGE profile analysis. Each color represents *vap1* profiles from one population. Bootstrap values refer to the similarity percentage of *vap1* patterns within and among populations.

Analyzing the gene variants using the PCR-DGGE procedure showed a difference in the *vap1* patterns among the studied populations of *H. schachtii (p* = 0.007, *d* = 76.28) (Table 1). There was a high similarity of *vap1* profiles among the replicates of the same population. The UPGMA clustering confirmed that each population has specific *vap1* variants (Figure 4).

Populations originating from different regions ordinated much closer than populations from the same one (Figure 4). Accordingly, the *vap1* similarities did not relate to their origin since similarity percentages were the highest between populations of different regions (Table 1).

## 3. Discussion

The genetically variable populations of *H. schachtii* originating from three German federal states differed in their biological characteristics. The main reason underlying these differences was the variability in the nematode and plant genes regulating biological processes of PPN, such as egg hatching, J2 attracting and penetration of the plant roots, forming the feeding sites, and thus the reproduction rate [14,17,18].

The weekly rate of hatching eggs was not uniform, and the ratio of the number of eggs hatched over eight weeks to the total egg content differed among the six populations. This result is consistent with a previous study that confirmed the difference in the hatching rate between a virulent population of *H. schachtii* and five others originating from different regions in the United States [1].

Studies indicate that various intrinsic and extrinsic factors govern the hatching process of PPNs [17]. In the present study, the internal factors are the only ones that affect the speed of egg hatching because the external conditions were uniform, such as the temperature and presence of the hatching solution (ZnCl_2_).

Among the internal reasons inhibiting egg hatching is incomplete embryonation [17]. To calculate the egg hatching percentage for *H. schachtii* populations, the number of hatched J2 to the total count of eggs containing embryonic juveniles was determined. Thus, embryo incompleteness cannot explain the difference in the hatching rate among populations.

The second internal factor is the activity of the juveniles inside eggs. For cyst nematodes, stylet thrusts to create a series of perforations, and eventually, a slit in the eggshell is essential for the emergency of pre-infective J2. Thrusts of J2 are preceded by an ion-mediated exchange of water and sugars across the eggshell, altering osmotic pressure, the flexibility of the eggshell, and the size of the egg [17,19]. For *H. schachtii*, this exchange can be spontaneous or due to a hatching stimulus such as plant secretions or ZnCl_2_ [20]. The difference in the speed of forming the slit in the eggshell might be a reason for the difference in the emergency rate of J2 among nematode individuals of *H. schachtii* populations.

PPNs respond to root exudate signals with host-specific gene expression [18]. The content of the root exudates affects the expression patterns of genes encoding the cell wall-degrading enzymes (CWDEs) secreted by the nematode-infective stages during the penetration and migration inside the host roots [18,21,22,23]. For example, the gene expression of *Pc-eng-1* and *Pc-xyl* in *Pratylenchus coffeae* correlates with cellulose and xylan quantities exuded by plant roots [18]. The penetration rate of one nematode population will therefore be variable among plant species that differ in the content of their root exudates.

In addition to the root emission, the variability of the plant wall compositions and protein receptors targeted by PPNs causes the plant–nematode interaction to occur in a host-specific manner. Polysaccharides, including cellulose and pectin, are the main components of the plant cell wall. The exact composition of polysaccharides is highly variable and depends on many factors, such as plant species, cell type, cell developmental stage, and external biotic and abiotic factors [24]. Further, the natural variation within papain-like cysteine protease Rcr3pim, which is the target of the cyst nematode effector VAP1, causes the difference in the plant–nematode interaction among the host cultivars [13,15,18]. In addition, the over-expression of plant pectin methyl esterase protein 3 (PME3), which interacts with the cellulose binding protein of *H. schachtii*, increased the nematode susceptibility in transgenic *Arabidopsis thaliana* [25,26]. These facts indicate that the nematode co-opts the host proteins for cell wall modification [25] and may clarify how the penetration and propagation ratios of each of the herein-studied populations of *H. schachtii* differed among the three cruciferous species.

Likewise, direct factors affecting the plant–nematode interaction are the genetic characteristics of the nematode population. A previous study stated that a population-specific gene expression in *H. glycines* exists before infection and during the onset of the parasitism process [27]. In a following study, parasitism genes encoding putative gland proteins were expressed differentially in pre-infective juveniles (pi-J2) between two populations of *H. glycines* [28]. The 1,4-Endoglucanase-coding gene encoding CWDE was also differentially expressed in J3 between those two populations [28]. The differences in the gene expression among *H. schachtii* populations are expected. That is because both nematode species of *H. schachtii* and *H. glycines* are closely related [1]. In addition, the flexibility in the structure of pectate lyases secreted by *H. schachtii* for root invasion and successive migration allows the protein to shrink or expand by the duplication or deletion of the DNA corresponding to one more β-strands [23,29]. In other words, the diversity of the genes encoding the pectate lyases might cause variation in the infection rate among individuals of *H. schachtii*.

The previous results confirmed the variability of the parasitism gene *vap1* within and among *H. schachtii* populations [5,7,13,14]. Consistent with those studies, the DGGE analysis of *vap1* fragments showed the difference in the gene pattern among the six studied populations of *H. schachtii*, referring to one of the putative factors causing the difference in the penetration ratio among those populations. The locus of the *vap1* gene analyzed in this study is composed of intron and exon regions [5]. The sequences of amino acids expressed from the exon regions are variable through the *vap1* variants [5], which could cause a functional change achieved by gene fragments that are variable among *H. schachtii* populations. To this point, the molecular marker of the effector gene *vap1*-based DGGE fingerprinting is efficient in distinguishing *H. schachtii* populations different in their biological characteristics.

All in all, the plant–nematode interaction between cruciferous plants and *H. schachtii* occurred in a host and population-specific manner. Since genetically different populations of *H. schachtii* are distinct in their pathogenicity, measures to control this nematode need to be modified accordingly. A population distinguished by its ability to propagate on resistant plants, the alteration among resistant hosts harboring different plant resistance genes can help to delay the propagation of virulent individuals and subsequently protect plant resistance against PPNs.

## 4. Materials and Methods

### 4.1. Nematode Materials

Based on previous results [7], which illustrated the gene patterns of *vap1* for *H. schachtii*, six nematode populations that differing in their *vap1* structures were selected. Those populations were derived from three regions in Germany, Lower Saxony (Berklingen and Holtensen), North Rhine Westphalia (Tizt-Kalrath and Vanikum), and Bavaria (Acholshausen and Brünnstadt), and reared on oilseed rape (*Brassica napus* ‘NK-fair’) for two generations.

To extract cysts, loess substrates of 100 g were washed with tap water in a bucket with a 250 µm sieve. The obtained cysts were collected on filter paper for the purposes described below.

### 4.2. Hatching Assay

The percentage ratio of hatched eggs to the egg content was calculated and compared among the six populations of *H. schachtii*. For each population, 500 cysts with three replicates were placed on a fleece filter in the hatching solution of ZnCl_2_ (408 mg/L) [30]. The juveniles that migrated downwards through the filter were collected and counted weekly. After the eighth week, the cysts were crushed to determine the number of unhatched eggs.

### 4.3. Bioassays to Estimate the Root Penetration and Propagation of H. schachtii Populations on Three Cruciferous Species

A greenhouse experiment at 20/16 °C for a 16/8 h day/night cycle was conducted to compare the penetration rate of six *H. schachtii* populations into roots of three plant species. One-week-old seedlings of cabbage (*Brassica oleracea* L., cv. Storema) or oilseed radish (*Raphanus sativus* L., cv. Siletina) or white mustard (*Sinapis alba* L., cv. Serval) planted in loess soil were inoculated with 450 J2 with eight replicates from each of the six populations. According to the descriptive list of cultivars published by the German Federal Plant Variety Office 2022, the Serval cultivar is in the third resistance class to *H. schachtii*. Six days after inoculation, the roots were harvested and stained with acid fuchsin [16]. The numbers of penetrated juveniles were evaluated and reported.

To compare the egg counts in the propagated cysts of the six populations, 14-day- old seedlings of the three plant species were inoculated with 450 J2 with eight replicates from each population. A total of 144 pots representing 18 treatments were placed under the abovementioned greenhouse conditions. After three months, corresponding to two generations of *H. schachtii*, plant shoots were cut off, and the pots containing the roots were left for two weeks to let the cysts mature. Soils were washed to extract the cysts following the published procedure of density centrifugation [16]. Counts of cysts and egg enumeration were estimated for each treatment.

### 4.4. Analyzing the vap1 Gene Variants of H. schachtii Populations

The published PCR-DGGE protocol was followed to characterize the *vap1* gene of the studied populations [5]. Briefly, for each population, the nematode DNA of 20 pooled cysts with four or five replicates was extracted using the proteinase k-lysis buffer. To amplify the *vap1* fragments from the genomic DNA, the primer pair HSvap244f/HSvap548rGC and the corresponding PCR conditions were applied. DGGE fingerprinting was conducted by loading 15 µL aliquots of PCR-amplified fragments on 6% polyacrylamide gel containing denaturing gradients of 40 to 58%.

The CIPHER electrophoresis system (C.B.S. Scientific, San Diego, CA, USA) was used to run the DGGE gel in 1x Tris-acetate-EDTA buffer at 58 °C for ten hours. DGGE gels were stained by GelStar^®^ Nucleic Acid solution (1x) (Rockland, ME, USA) using the manufacturer’s protocol and visualized under the UV transilluminator.

### 4.5. Data Analysis

The R statistical 4.0.4 software packages were applied to analyze the data related to the counts of penetrated J2 and propagated cysts. GelCompar II 6.5 software (Applied Math, Gent, Belgium) was utilized to create a similarity matrix based on the Jaccard coefficient from DGGE banding patterns of *H. schachtii* populations. The PERMTEST [31] was run to compare the *vap1* similarity measures.

## 5. Conclusions

Analyzing the population genetic structures is essential to explain differences in the pathogenicity among nematode populations. Genetic characterization also paves the way toward the protection of plant resistance. For example, cultivating a resistant variety to suppress a field population highly genetically similar to the standard population will avoid the breakdown of plant resistance. Moreover, monitoring the parasitism gene diversity in nematode populations could be a powerful approach to provide a way to select plant genes toward durable resistance against PPNs. Finally, resolving the population genetic structure of the target PPNs plays a pivotal role in understanding the mechanisms of the resistance breakdown and subsequently improving the effectiveness of resistance breeding.

## Figures and Tables

**Table 1:** Similarities of the *vap1* patterns among six populations of *H. schachtii* originating from three geographic regions (Berklingen and Holtensen/Lower Saxony; Titz-Kalrath and Vanikum/North Rhine Westphalia; Acholshausen and Brünnstadt/Bavaria).

Compared Populations	*vap1* Similarity	*p*-Value
Berklingen-Holtensen	13.48%	0.02 *
Berklingen-Titz-Kalrath	23.33%	0.02 *
Berklingen-Vanikum	20.47%	0.03 *
Berklingen-Acholshausen	35.91%	0.0 *
Berklingen-Brünnstadt	16.59%	0.02 *
Holtensen-Titz Kalrath	11.11%	0.02 *
Holtensen-Vanikum	65.88%	0.02 *
Holtensen-Acholshausen	7.9%	0.0 *
Holtensen-Brünnstadt	24.99%	0.02 *
Titz Kalrath-Vanikum	18.68%	0.02 *
Titz Kalrath-Acholshausen	34.27%	0.0 *
Titz Kalrath-Brünnstadt	20.52%	0.02 *
Vanikum-Acholshausen	19.45%	0.0 *
Vanikum-Brünnstadt	21.65%	0.02 *
Acholshausen-Brünnstadt	14.1%	0.0 *

* Refers to the significance calculated by permutation test on pairwise similarities based on Jacquard coefficient.

## Data Availability

Data are available from the corresponding author on reasonable request.
